# Carotenoid accumulation during tomato fruit ripening is modulated by the auxin-ethylene balance

**DOI:** 10.1186/s12870-015-0495-4

**Published:** 2015-05-08

**Authors:** Liyan Su, Gianfranco Diretto, Eduardo Purgatto, Saïda Danoun, Mohamed Zouine, Zhengguo Li, Jean-Paul Roustan, Mondher Bouzayen, Giovanni Giuliano, Christian Chervin

**Affiliations:** Université de Toulouse, INP-ENSA Toulouse, UMR990 Génomique et Biotechnologie des Fruits, Avenue de l’Agrobiopole, CS 32607, F-31326 Castanet-Tolosan, France; INRA, UMR990 Génomique et Biotechnologie des Fruits, 24 Chemin de Borde Rouge, CS 52627, F-31326 Castanet-Tolosan, France; Italian National Agency for New Technologies, Energy, and Sustainable Development, Casaccia Research Centre, 00123 Rome, Italy; Department Food and Experimental Nutrition; NAPAN/FoRC - Food Research Center, Universidade de São Paulo, School of Pharmaceutical Sciences, Av. Prof. Lineu Prestes 580, Butantã, CEP 05508-000 São Paulo, SP Brazil; Université de Toulouse; UPS; UMR 5546; Laboratoire de Recherche en Sciences Végétales (LRSV), 24 Chemin de Borde Rouge, F-31326 Castanet-Tolosan, France; Genetic Engineering Research Centre, Bioengineering College, Chongqing University, Chongqing, 400044 PR China; Actual address: Department of Life Sciences, Xi’an University of Arts and Science, Xi’an, 710065 PR China

**Keywords:** Auxin, Ethylene, Abscisic acid, Tomato, Carotenoids, Chlorophyll, Lycopene, Rin, Ripening

## Abstract

**Background:**

Tomato fruit ripening is controlled by ethylene and is characterized by a shift in color from green to red, a strong accumulation of lycopene, and a decrease in β-xanthophylls and chlorophylls. The role of other hormones, such as auxin, has been less studied. Auxin is retarding the fruit ripening. In tomato, there is no study of the carotenoid content and related transcript after treatment with auxin.

**Results:**

We followed the effects of application of various hormone-like substances to “Mature-Green” fruits. Application of an ethylene precursor (ACC) or of an auxin antagonist (PCIB) to tomato fruits accelerated the color shift, the accumulation of lycopene, α-, β-, and δ-carotenes and the disappearance of β-xanthophylls and chlorophyll *b*. By contrast, application of auxin (IAA) delayed the color shift, the lycopene accumulation and the decrease of chlorophyll *a*. Combined application of IAA + ACC led to an intermediate phenotype. The levels of transcripts coding for carotenoid biosynthesis enzymes, for the ripening regulator *Rin,* for chlorophyllase, and the levels of ethylene and abscisic acid (ABA) were monitored in the treated fruits. Correlation network analyses suggest that ABA, may also be a key regulator of several responses to auxin and ethylene treatments.

**Conclusions:**

The results suggest that IAA retards tomato ripening by affecting a set of (i) key regulators, such as *Rin*, ethylene and ABA, and (ii) key effectors, such as genes for lycopene and β-xanthophyll biosynthesis and for chlorophyll degradation.

**Electronic supplementary material:**

The online version of this article (doi:10.1186/s12870-015-0495-4) contains supplementary material, which is available to authorized users.

## Background

Auxin and ethylene are hormones known to impact plant development, often with antagonistic roles. Auxin exerts pleiotropic effects, on the development of roots, shoots, flowers and fruits [1]. Ethylene is one of the plant hormones regulating the ripening of fruits, the opening of flowers, and the abscission of leaves. Tomato is a model plant for the study of climacteric fruit development, which is promoted by ethylene [2]. Observations of tomato fruits and some non-climacteric fruits, like grape berry and strawberry, have suggested that ripening is also regulated by auxin, since they can delay ripening and regulate gene expression [3-6]. However, the impact of auxin on tomato ripening has not been extensively studied, as previous works using exogenous auxin [3,6] do not study carotenoid accumulation and related gene expression. Moreover in the plant kingdom, the crosstalk between auxin and ethylene is not yet deciphered [7].

**Figure 1:**
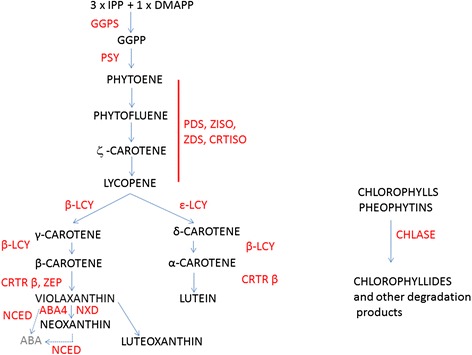
Carotenoid biosynthetic pathway based on a previous study [12]. Names of intermediate compounds are in black and names of enzymes are in red. IPP = isopentenyl diphosphate, GGPS = GGPP synthase, GGPP = geranyl-geranyl pyrophosphate, PSY = phytoene synthase, PDS = phytoene desaturase, ZISO = zeta-carotene isomerase, ZDS = zeta-carotene desaturase, CRTISO = carotenoid isomerase, ε-LCY = lycopene ε-cyclase, β-LCY = lycopene β-cyclase, CRTR-β = β-carotene hydroxylase, ZEP = zeaxanthin epoxydase, NXD = neoxanthin synthase, CHL = chlorophyllases, ABA = abscisic acid.

Color change from green to red is a very important indicator of tomato ripening and can easily be measured by chromametry [8]. This change is associated with the degradation of chlorophylls and the shift of the carotenoid composition from leaf-like xanthophylls (mainly lutein and neoxanthin) to carotenes (mainly phytoene, lycopene and β-carotene) as described by Fraser et al. [9]. In the fruit tissues, the degradation of chlorophylls is slow, while the accumulation of red carotenoids is rapid [10] when checked by time lapse imaging. The carotenoid biosynthetic pathway in tomato is well described [11,12] and is detailed on Figure 1. The first committed step is the condensation of two molecules of geranylgeranyl diphosphate (GGPP) to form the colorless carotene 15-*cis*-phytoene, a reaction catalyzed by phytoene synthases (PSY); 15-*cis*-phytoene is then desaturated and isomerized to all-*trans*-lycopene through the action of two desaturases and two isomerases: phytoene desaturase (PDS), ζ-carotene desaturase (ZDS), prolycopene isomerase (CRTISO) and ζ-carotene isomerase (ZISO). The formation of δ-carotene and γ-carotene from lycopene are catalyzed by lycopene ε-cyclase (ε-LCY) and β-cyclases (β-LCY and CYC-β), and then the orange α - carotene and β-carotene are synthetized by β-cyclases. Finally, these carotenes are transformed into lutein and zeaxanthin by heme and non-heme β-carotene hydroxylases (CYP97 and CRTR-b). Zeaxanthin is converted to violaxanthin by the action of zeaxanthin epoxidase (ZEP) and further to neoxanthin by the action of the NXD and ABA4 proteins. These two xanthophylls are cleaved by 9-*cis*-epoxycarotenoid dioxygenase (NCED), a key enzyme in the biosynthesis of ABA [13].

For the purpose of this article, the pathway will be divided into two parts, upstream of lycopene and downstream of lycopene (Figure 1). In the upstream part, the key rate-limiting steps are catalyzed by PSY1, PDS, ZDS, ZISO and CRTISO [9,14,15]. The expression of *Psy1, Ziso, Crtiso* is directly regulated by the ripening inhibitor (RIN) protein, which is a member of the MADS-box family of transcription factors [16,17]. In the downstream part, lycopene cyclases (ε-LCY, β-LCY/CYC-β) are also key enzymes, catalyzing the transformation of lycopene to δ- and β- carotene [18-21].

To study the role of cross-talk between auxin and ethylene in the accumulation of carotenoid pigments in tomato fruits, we treated mature green fruits with the auxin indole acetic acid (IAA) and the ethylene precursor aminocyclopropane carboxylic acid (ACC), alone or in combination, and also with p-chlorophenoxy isobutyric acid (PCIB). The latter compound is an antagonist of auxin action, although its mechanism of action is not well characterized [22]. The effects of these treatments on color change, pigment content and on the levels of transcripts involved in carotenoid biosynthesis were studied.

## Results and Discussion

### Contrasting effects of ethylene and auxin on tomato fruit color

**Figure 2:**
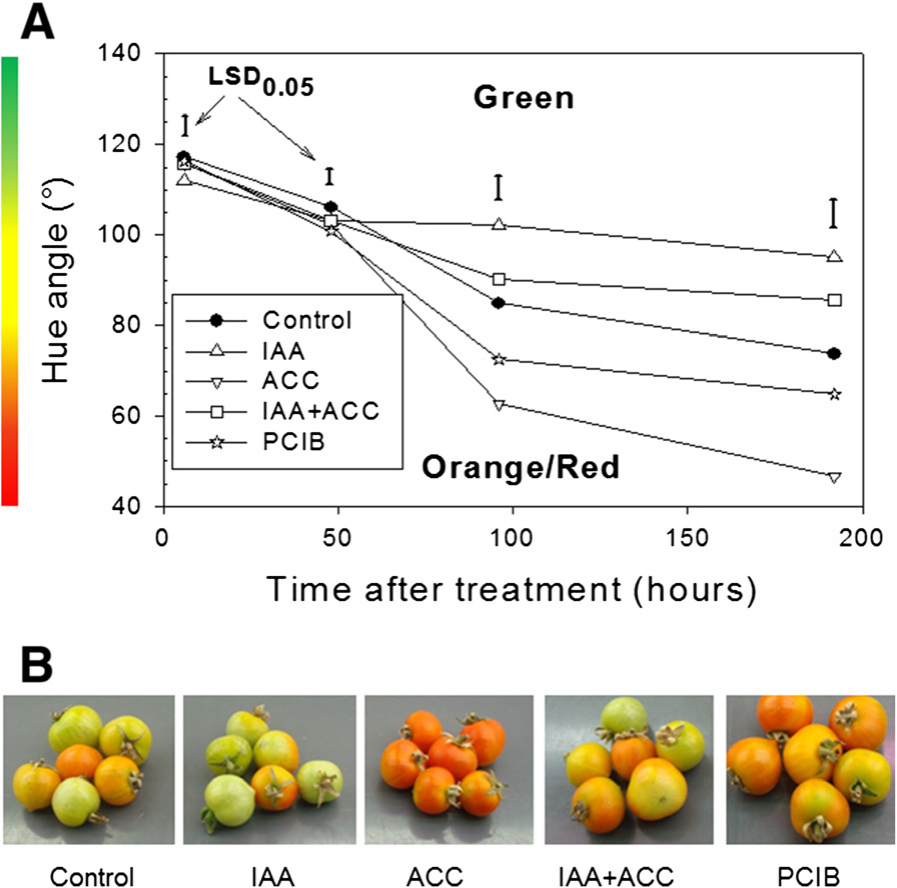
**A)** Changes of tomato color as a function of time after hormonal treatments. IAA: indole acetic acid, ACC: aminocyclopropane carboxylic acid (ethylene precursor), PCIB: p-chlorophenoxy isobutyric acid (auxin antagonist). The color bar next to the Y axis gives an indication of the relation between Hue angle and fruit color, but it is not the exact color of the fruit on the CIELab scale. n = 6 biological replicates, LSD bars calculated at 0.05 level. **B)** Pictures of tomatoes 96 h after hormonal treatments.

The hormonal treatments induced significant color changes within 96 hours (Figure 2). Treatment with ACC accelerated significantly the transition from green to orange/red compared to controls. On the contrary, treatment with IAA induced a significant delay in the transition from green to orange/red compared to controls. After 96 h, IAA-treated fruits began to turn orange and then never became red (data not shown).

In fruits treated with a combination of ACC and IAA, color evolution was slower than in controls, but faster than the fruits treated by IAA alone, indicating that IAA treatment is epistatic over ACC treatment. In the presence of the auxin antagonist PCIB, fruits turned red faster than control ones and the color change kinetics were very similar to those treated with ACC (Figure 2A). These results confirmed previous studies showing that IAA slows down ripening of tomato fruits [2,6], and that ACC accelerates it [2].

### Effects of hormonal treatments on carotenoid, chlorophyll and ABA accumulation

**Figure 3:**
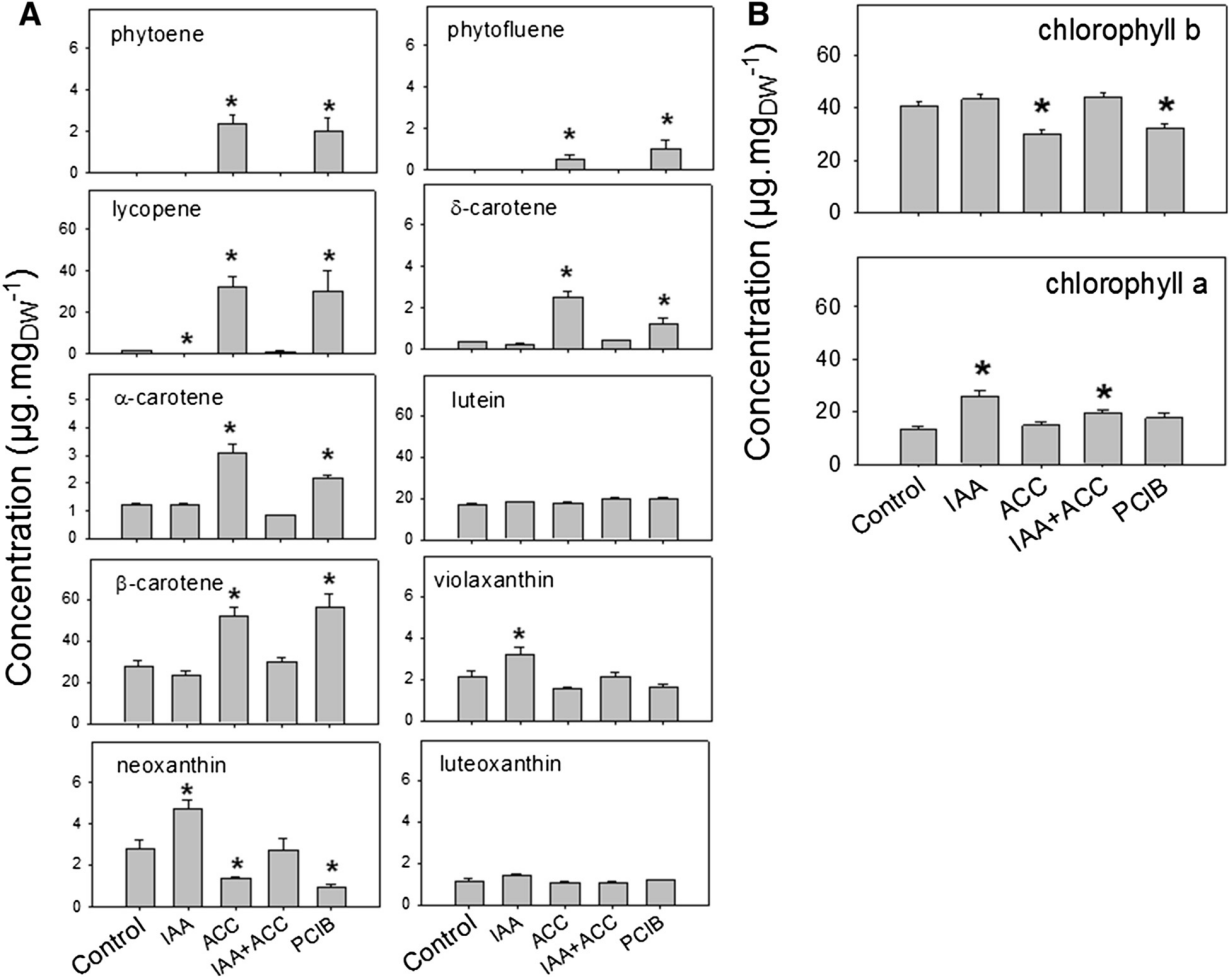
Carotenoid **[A]** and chlorophyll **[B]** contents 96 h after hormonal treatments. Abbreviations are as in Figure 2. n = 3 biological replicates, error bars are standard errors. An asterisc (*) shows a significant difference at 0.05 level using t-test between control and the corresponding treatment.

To further investigate the influence of hormonal treatments on fruit pigment composition, fruit extracts were analyzed. At 96 hours, the main carotenoids in control fruits were lutein and β-carotene (Figure 3). Large amounts of chlorophylls *a* and *b* were observed, together with trace amounts of lycopene, violaxanthin, neoxanthin, luteoxanthin, ζ-, δ- and α-carotene. The upstream compounds phytoene and phytofluene were not detectable. This composition is typical of a ripening stage between the “Breaker” and “Orange” stages of ripening [41].

The ACC and PCIB treatments induced large changes in carotenoid composition at 96 hours (Figure 3). Lycopene was greatly induced, becoming a major pigment, together with β-carotene which was also induced and lutein which was unaffected. The upstream compounds phytoene, phytofluene and ζ-carotene and the downstream compounds δ- and α-carotene were also induced, while the β-xanthophylls, neoxanthin and violaxanthin were reduced.

The IAA treatment reduced significantly lycopene accumulation compared to controls while it did not affect α-, β- or δ-carotene accumulation. It also led to higher levels of neoxanthin, violaxanthin and chlorophyll *a* than in the controls (Figure 3).

**Figure 4:**
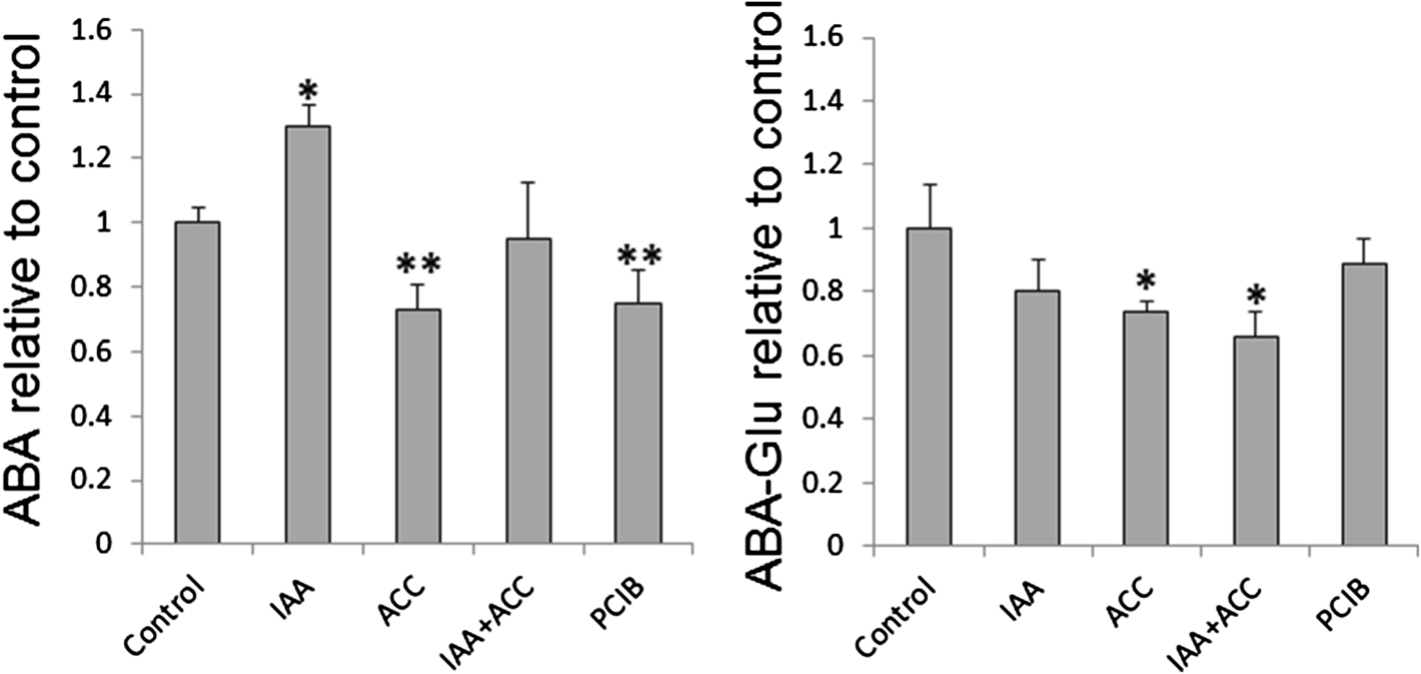
Variations of free ABA levels and ABA glucoside 96 h after hormonal treatments. Abbreviations are as in Figure 2. n = 5 biological replicates, error bars are standard errors. Asteriscs, * or ** show significant differences at the 0.05 or 0.01 levels compared to controls, respectively (t-test).

The 9-*cis* forms of neoxanthin and violaxanthin are the precursors of abscisic acid (ABA) [23,24], a phytohormone known to control ripening of many fruits, including tomato, in which it triggers ethylene biosynthesis and thus accelerates ripening [25]. ABA levels were decreased by the ACC and PCIB treatments and increased by the IAA treatment (Figure 4), mimicking the evolution of neoxanthin/violaxanthin, thus suggesting that the accumulation of these compounds might be directly correlated. This observation is consistent with the idea that in the tomato fruit, levels of neoxanthin and violaxanthin are rate-limiting for ABA accumulation [26]. Finally, the ACC and PCIB treatments led to an increased degradation of chlorophyll *b* (Figure 3).

Our results detail the auxin effects on carotenoid accumulation, thus completing preliminary observations that were not detailing this aspect [6]. Our results also detail carotenoid changes induced by ACC, following previous studies showing that ethylene treatments accelerated chlorophyll degradation, the appearance of orange color [10,27] and the accumulation of lycopene [28]. It is noticeable that PCIB, which acts as an auxin antagonist, induced the same effects as ACC.

### Effects of hormonal treatments on gene expression

**Figure 5:**
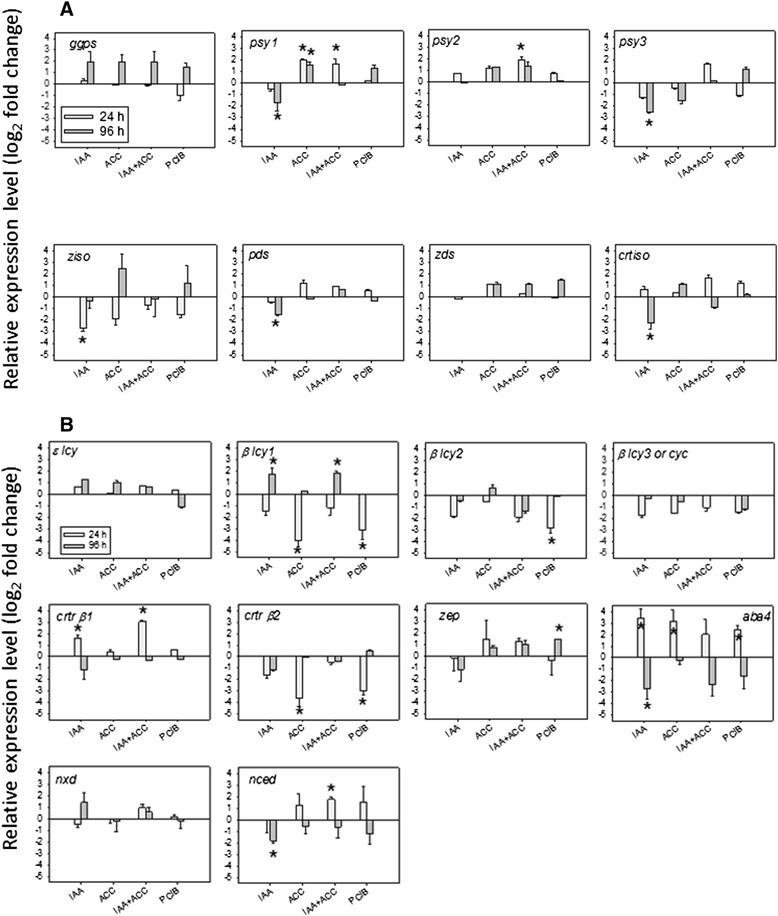
Modulation of transcript accumulation related to carotenoid pathway, **A)** upstream of lycopene, **B)** downstream of lycopene, 24 h or 96 h after hormonal treatments (see abbreviations in legend of Figure 2). n = 3 biological replicates, bars = std errors. Expression relative to controls (set at 0). An asterisc (*) shows significant differences at the 0.05 level with controls (t-test).

In order to investigate if the above hormone-induced phenotypes were controlled at least partially at the gene expression level, we determined the levels of all transcripts involved in carotenoid biosynthesis by quantitative Real Time PCR (qPCR) at two different times after the hormonal treatments (Figure 5).

As observed in Figure 5A, IAA treatment resulted in lower transcript levels for most of the genes upstream of lycopene (*Psy1, Psy3*, *Pds, Ziso* and *Crtiso*). With the exception of *Psy3* which has been reported to be mainly expressed in roots, all these genes are rate-limiting for lycopene accumulation [15]. Thus, these changes in transcript levels match well the slower color change and the decreased accumulation of lycopene after treatment with IAA (Figures 2 and 3). Regarding the downstream part of the pathway (Figure 5B), the transcript levels of *β-Lcy1* and *Crtr-β1* genes were induced by IAA treatment, concomitant with the higher amounts of violaxanthin and neoxanthin, while *Aba4* showed a biphasic response (induction at 24 h and repression at 96 h) and *Nced1* a repression at 96 h. Together, these observations indicate that the ABA increase after IAA treatment is a fast response, probably due to an increase in the synthesis of its precursors violaxanthin and neoxanthin, mediated by an activation of the *β-Lcy1*, *Crtr-β1* and *Aba4* genes. The repression of *Aba4* and *Nced1* at 96 h may be due to a negative feedback regulation exerted by the increased ABA levels on these genes. ABA is known to increase in tomatoes prior to the ethylene peak [25].

ACC treatment led to higher levels of *Psy1* and *Psy2* transcripts*,* and also*,* to a lesser extent, of the *Ziso, Pds, Zds* and *Crtiso* ones (Figure 5A). All these genes encode rate-limiting steps for lycopene biosynthesis [15] and thus the observed changes in gene expression are in agreement with the faster color change and accelerated lycopene accumulation (Figures 2 and 3). Moreover, ACC treatment decreased *β-Lcy1* transcript levels (Figure 5B) with unexpected increase of α-, β- and δ- carotenes, indicating that the *β-Lcy1* repression was possibly offset by the unaltered levels of the other cyclase transcripts. ACC also repressed *Crtr-β2* expression that was not offset by the unaltered *Crtr-β1* levels, reducing the further conversion of carotene compounds into β-xanthophylls. This was confirmed by the reduced neoxanthin and ABA levels after ACC treatment (Figures 3 and 4), in spite of an induction of *Aba4*. It is also worth noticing that IAA and ACC affected the expression of two different hydroxylase paralogs, *Crtr-βi* being stimulated by IAA and *Crtr-β2* being inhibited by ACC, respectively. Overall, these data explain the faster accumulation of lycopene and β-carotene, and also the lower accumulation of β-xanthophylls and ABA in ACC treated fruits than in controls.

Similar changes in transcript levels occurred in PCIB-treated fruits (Figure 5), which showed an additional repression of *β-Lcy2* and an induction of *Zep,* as well as a very similar carotenoid profile (Figure 3) to the ACC-treated samples. There was no significant effect of any treatment on *Ggps* expression (Figure 5A and Additional file 1: Figure S1).

The combined IAA + ACC treatment resulted in a visual and carotenoid phenotype intermediate between those of each treatment alone and more similar to that of IAA alone, with the exception of violaxanthin, neoxanthin and ABA induction, which was less pronounced than in IAA alone (Figures 2, 3 and 4). At the transcriptional level, IAA + ACC was less inhibitory of upstream transcripts than IAA alone. Although the significance of these observations awaits clarification, it confirms the antagonistic effects of the two hormones at the biochemical and transcriptional levels.

**Figure 6:**
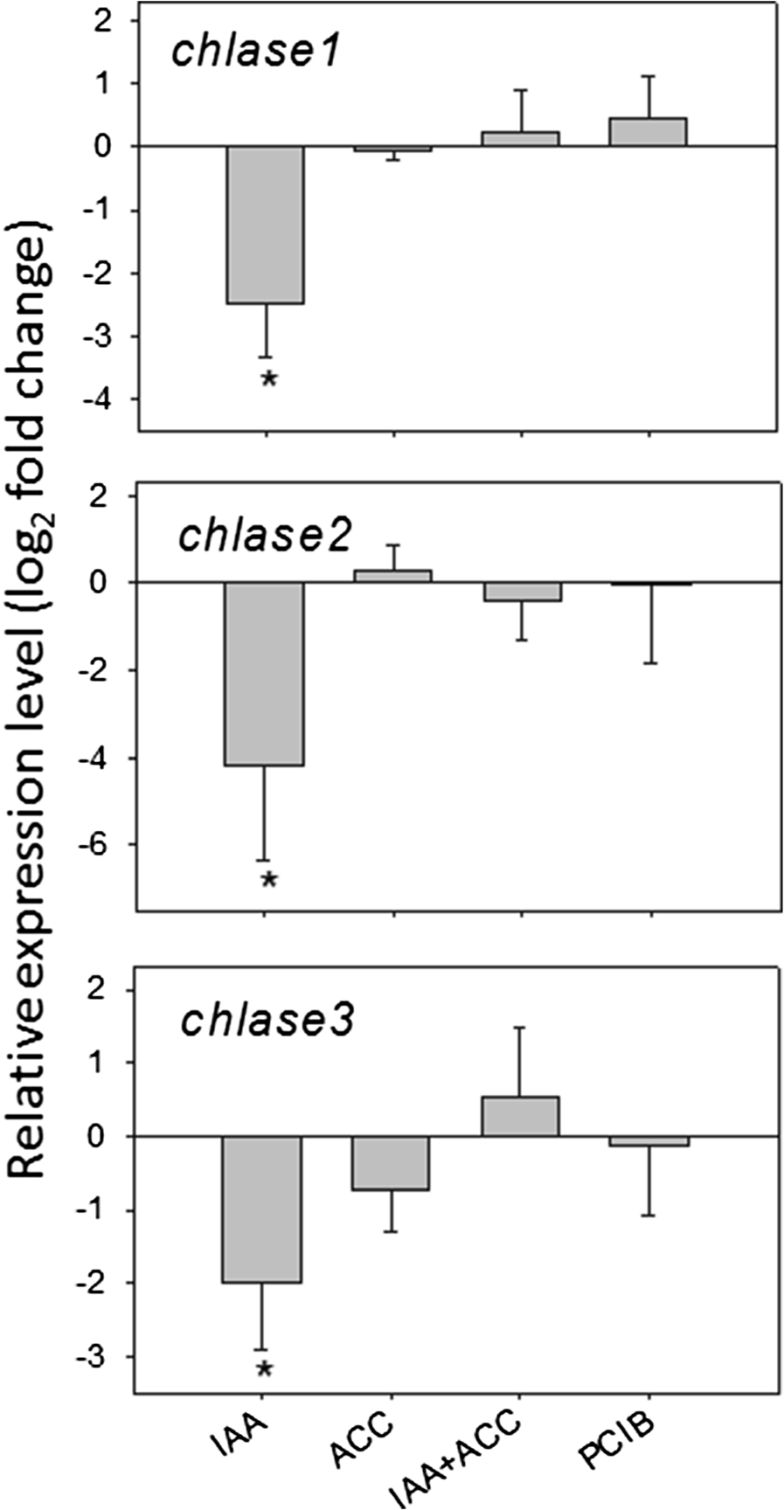
Modulation of chlorophyllase transcripts, 96 h after hormonal treatments (see abbreviations in legend of Figure 2). n = 3 biological replicates. Expression relative to controls (set at 0). Error bars are standard errors. An asterisc (*) shows significant differences the 0.05 level with controls (t-test).

Chlorophyll degradation in *Citrus* fruits is an active process mediated by chlorophyllase (Chlase) [29]. In tomato, chlorophyll degradation was affected by hormonal treatments, with IAA treatment retarding chlorophyll *a* degradation, both alone and in combination with ACC treatment, while chlorophyll *b* degradation was accelerated by both ACC and PCIB treatments (Figure 3). We measured the levels of the three *Chlase* transcripts identified in the tomato genome. Repression of all three transcripts was obvious 96 h after the IAA treatment (Figure 6). This correlates well with the higher levels of chlorophyll *a* and to a lesser extent of chlorophyll *b,* in both treatments with IAA (Figure 3). However, the marked decrease of chlorophyll *b* in the ACC and PCIB treatments does not correlate with increased *Chlase* transcript accumulation (Figure 6). This suggests that, in contrast to Citrus [29], tomato *Chlase* gene expression is not under ethylene control and that, as observed in Citrus [30], posttranscriptional mechanisms may also regulate *Chlase* activity in tomato.

### Effects of hormonal treatments on the Rin transcript and on transcripts of the carotenoid/ABA pathway

**Figure 7:**
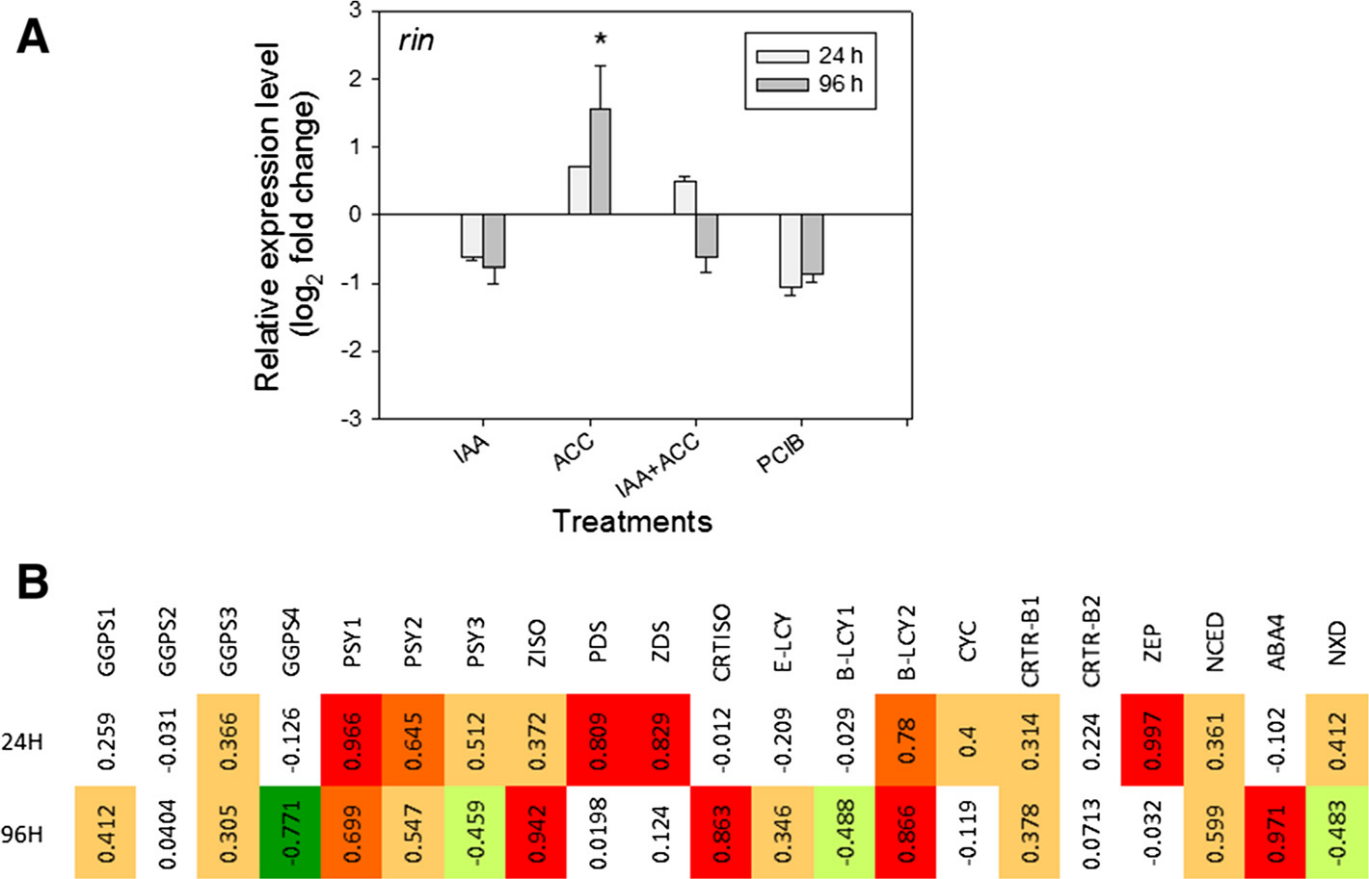
**A)** Modulation of the Rin transcript 24 h or 96 h after various hormonal treatments (see abbreviations in legend of Figure 2). n = 3 biological replicates. Expression relative to controls (set at 0). Error bars are standard errors. **B)** Correlation coefficients between Rin and other transcripts shown in Figure 5.

Several genes in the carotenoid pathway are regulated by the Rin transcription factor [16,17]: *Psy1*, *Ziso* and *Crtiso* display direct positive regulation, *Zds* indirect positive regulation, and *ε-Lcy* and *β-Lcy2* indirect negative regulation. Analyses carried out by qPCR (Figure 7A) showed that the transcript levels of *Rin* were stimulated by ACC and inhibited by IAA, even if the sole significant difference was noticed for ACC 96 h. The qPCR profiles of *Rin* (Figure 7A) and *Psy1* (Figure 5A) seem to match quite well. Indeed, in keeping with the findings of Fujisawa et al. [17], high positive correlations (ρ > 0.60, and in some cases ρ > 0.80) were observed between transcript levels of *Rin* and *Psy1* at both 24 h and 96 h, *Ziso* and *Crtiso* at 96 h, and *ZDS* at 24 h (Figure 7B).

In contrast, *ε-Lcy* did not show high correlations with *Rin* neither at 24 h nor at 96 h, while *β-Lcy2* showed strong positive correlations at both time points. This contrasts with the findings of Fujisawa et al. [17] and suggests that lycopene cyclase transcripts are subject to additional layers of regulation. Strong positive correlations with *Rin* were identified for *Pds* and *Zep* at 24 h and for *ABA4* at 96 h. The latter two genes mediate the biosynthesis of the ABA precursors, violaxanthin and neoxanthin (Figure 1), and thus their positive correlations with *Rin* may be indicative of the fact that Rin activates two hormonal cascades: one acting through ethylene [16], and one acting through ABA. Finally, *Ggps4* showed a negative correlation with *Rin* levels at 96 h. This gene is unrelated to fruit carotenoid biosynthesis and may control the biosynthesis of other isoprenoid compounds (Falcone et al., unpublished).

### Effects of hormonal treatments on fruit ethylene production

**Figure 8:**
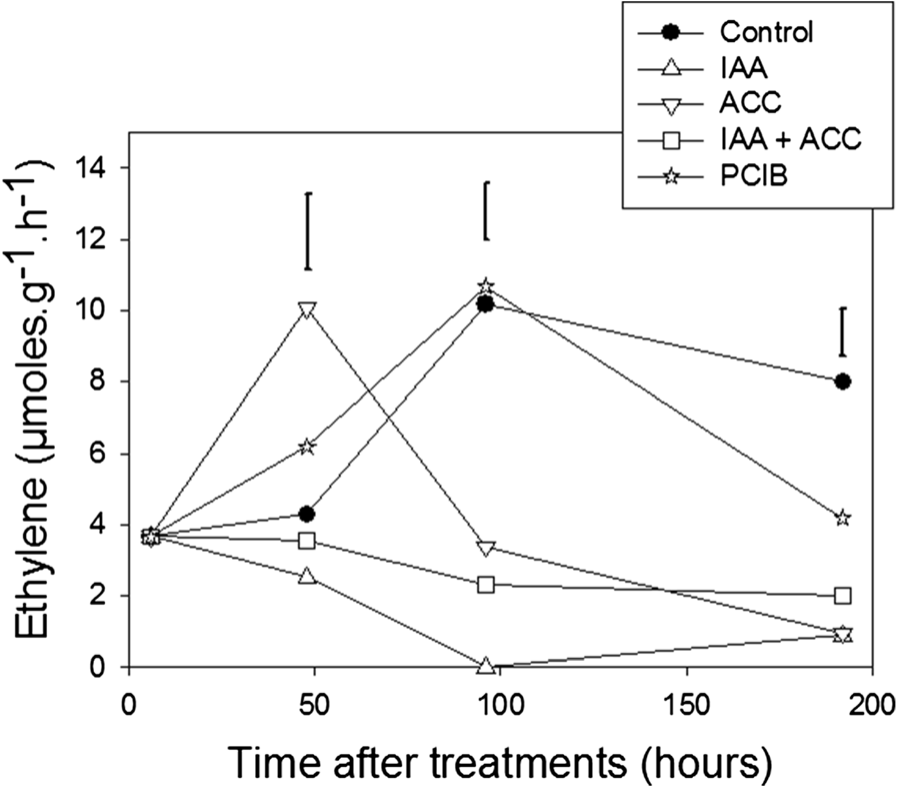
Variations in ethylene production after the hormonal treatments (see abbreviations in legend of Figure 2). n = 3 biological replicates, error bars are LSD at the 0.05 level.

Ethylene is assumed to be a “master switch” controlling tomato fruit ripening. Therefore, it is interesting to verify if the hormonal treatments described above alter ethylene production. We measured ethylene production in hormone-treated fruits at various times after treatments (Figure 8). As expected, ACC treatment accelerated the appearance of the climacteric ethylene peak by about 2 days whereas IAA treatment repressed the ethylene production, and this repression was only partially reversed by combined IAA + ACC treatments. PCIB treatment had little effect up to 100 hours after treatment, while it slightly decreased ethylene production around 200 hours. So it seems that PCIB enhancement of carotenoid accumulation in comparison to controls (Figure 2) is not mediated by a variation in ethylene production. The IAA decrease of carotenoid accumulation in comparison to controls could be partially mediated by the repression of ethylene production.

### Factorial and network analyses show associations between hormonal treatments and carotenoid levels

**Figure 9:**
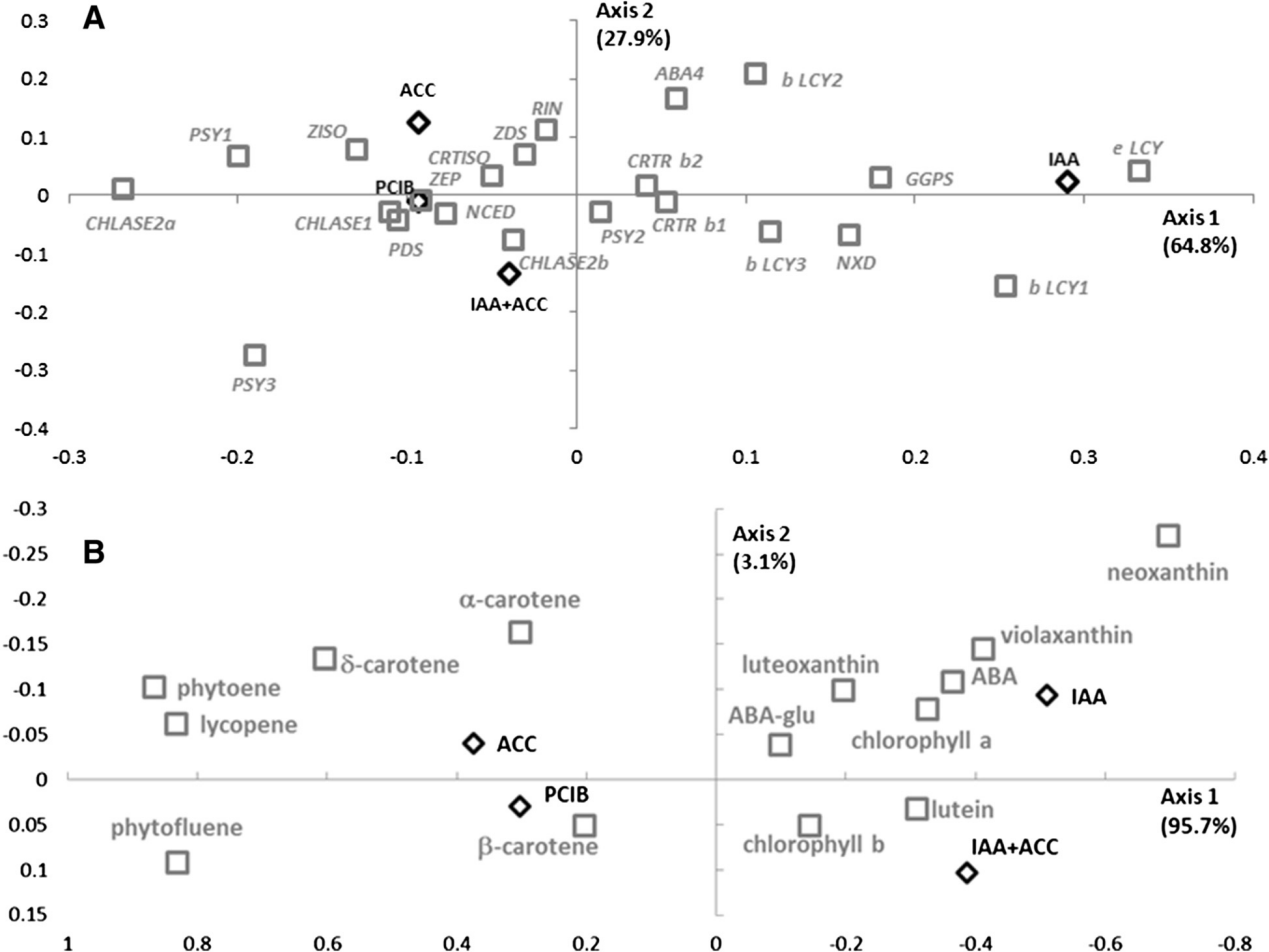
Factorial correspondence analyses with data 96 h after hormonal treatments, **A)** transcript accumulation and **B)** carotenoid and metabolite content. Abbreviations are as in Figures 1 and 2.

Factorial analyses are used to determine and describe the dependencies within sets of variables. In this study the treatments, and many observed variations, in this study the transcript levels (Figure 9A) or the carotenoid levels (Figure 9B). These factorial correspondence analyses clearly show strong positive correlation between the effects of ACC and PCIB, and their negative correlation to the effects of IAA treatment, whatever the regulatory level measured: transcript accumulation or carotenoid accumulation. It is noticeable that, at the transcript level, the IAA + ACC treatment is strongly correlated with the ACC and PCIB ones (Figure 9A), while at the carotenoid composition level - which matches the fruit phenotype more closely - it is correlated with the IAA treatment (Figure 9B). This may be due to the fact that changes in transcript accumulation occur ahead of those in metabolite accumulation, or to the fact that some of the latter changes are due to post-transcriptional events, or to both.

The transcripts correlating with the ripening delay associated to IAA treatment are lycopene cyclases (*ε and β-Lcy*) and, to a lesser extent, carotene hydroxylases (*Crtr-β*) (Figure 9A). These results confirm previous studies [18-21]. All transcripts mediating lycopene biosynthesis in tomato fruits: *Psy1, Pds, Ziso, Zds*, and *Crtiso* [15] correlate well with the accelerated ripening induced by ACC or PCIB. Also *Psy3*, which is much less expressed and is non-essential for lycopene biosynthesis, shows a position opposed to IAA treatment (Figure 9A) as it was strongly repressed by IAA at 96 h (Figure 5A). Same case for the position of *Chlase* transcripts in Figure 9A which is mainly due to the strong inhibition by IAA, rather than to a stimulation by ACC. Regarding carotenoids, the accumulation of upstream intermediates and lycopene and, to a lesser extent, of α-, β-, and δ-carotene is correlated directly with ACC and PCIB treatments. Inversely IAA and IAA + ACC treatments correlate well with chlorophylls and xanthophylls, (especially violaxanthin and neoxanthin) and their product ABA (Figure 9B). This is consistent with the fact that ripening is associated with the accumulation of cyclic carotenes and with the decrease of chlorophylls and xanthophylls.

**Figure 10:**
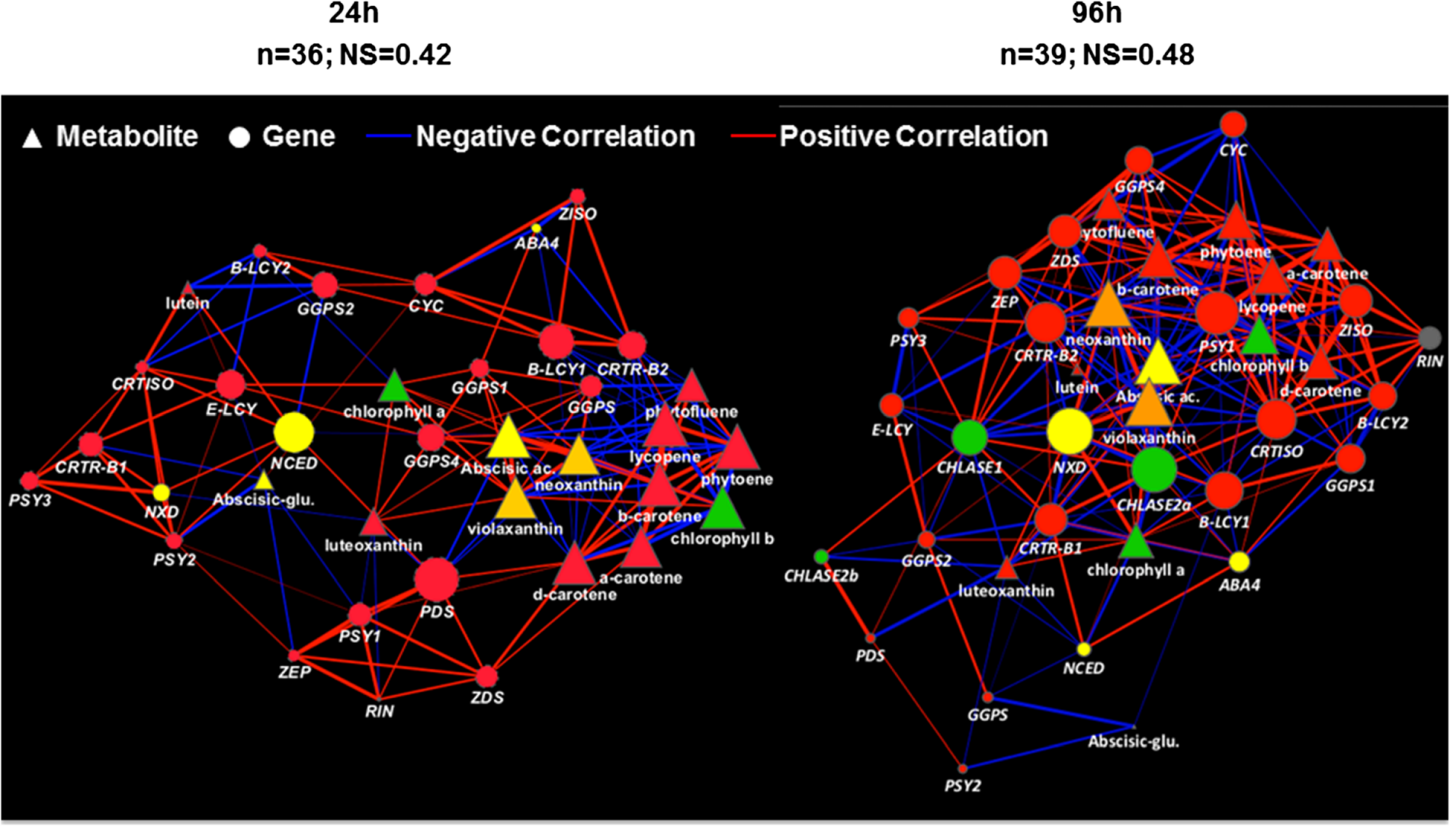
Correlation networks at 24 h and 96 h, generated as previously described [31]. In all network diagrams, nodes of different shape represent genes and metabolites. Direct and inverse corrlations ≥ |0.60| are shown as red and blue edges, respectively. Edge thickness is proportional to the absolute values of the Pearson correlation coefficient (|ρ|), while node sizes are proportional to node strengths [31] (Additional file 2: Table S2). n = number of nodes, NS = network strength [31]. Nodes related to carotenoids are shown in red, to chlorophyll in green, to ABA in yellow, neoxanthin and violaxanthin (ABA precursors) in orange, Rin in grey. The “organic layout” was used for network visualization with Cytoscape 2.6.3 (www.cytoscape.org) [32].

We also applied correlation network analysis based on transcript-metabolite data integration (Figure 10). The time spent after treatments increased the strength in the network [31], and at 96 h the network shows four nodes with strong correlation values (|ρ| > 0.60) (Additional file 2: Table S2): ABA, its metabolic precursors violaxanthin and neoxanthin and *Nxd,* a gene essential for neoxanthin biosynthesis [33]. All four nodes exhibited a prevalence of negative correlations with the other ripening-specific variables in the network.

## Conclusions

Our results suggest that ACC treatment induces lycopene and α-, β- and δ-carotene accumulation by inducing *Psy1*, and repressing *β-Lcy1* and *Crtr-β2.* These transcriptional responses are fast, reaching a peak at 24 h*.*

On the other hand, treatment with IAA represses several upstream carotenoid transcripts (*Psy, Ziso, Pds, Crtiso*) as well as *Chlases 1-3* and promotes the accumulation of *β-Lcy1* and *Crtr-β1* transcripts, leading to higher levels of chlorophyll *a*, neoxanthin, violaxanthin and ABA*.* These responses show a temporal curve: *Ziso* and some downstream transcripts (*Crtr-β1* and *ABA4*) respond already at 24 h, while most other transcripts (*Psy1* to *β-Lcy1*) respond later, at 96 h. This response could be due to the fact that downstream transcripts respond directly to auxin, while upstream transcripts respond to the repression of ethylene production induced by IAA treatment (Figure 8). Treatment with PCIB (an auxin antagonist) led to responses similar to those obtained after ACC treatment, confirming the antagonism between ethylene and auxin. Interestingly, while IAA completely repressed ethylene production, PCIB did not increase it (Figure 8) indicating that endogenous auxin does not play a major role in regulating ethylene production during normal ripening. The repression of ethylene production and the induction of *Crtr-β1* by exogenous IAA supplementation were epistatic over ACC supplementation when both treatments were given together, while the final phenotype of the fruits did not show a clear epistasis of IAA over ACC supplementation.

Factorial and correlation network analyses allowed the identification, at 96 h, of transcriptional and metabolite “hubs” which may represent central regulators; these comprised ABA, its carotenoid precursors (violaxanthin and neoxanthin) and the *Nxd* gene, leading to neoxanthin biosynthesis. Overall, these data suggest a central role for ABA as a negative intermediate regulator in the perturbation of tomato fruit ripening following auxin and ethylene treatments.

## Methods

### Plant materials and growth conditions

Tomato plants (*Solanum lycopersicum* cv. *MicroTom*) were grown under standard greenhouse conditions. The culture chamber room was set as follows: 14-h day/10-h night cycle, 25/20°C day/night temperature, 80% relative humidity, 250 μmol m^-2^ s^-1^ light intensity. Tomato seeds were first sterilized 5 min in sterile water and sown in Magenta vessels containing 50 ml 50% Murashige and Skoog (MS) culture medium and 0.8% (w/v) agar, pH 5.9 [34].

### Treatments of tomato fruits

Tomato fruits were harvested at the mature green stage of development and injected with a buffer solution contained 10 mM MES, pH 5.6, sorbitol (3% w/v) and 100 μM of ACC, or IAA, or IAA + ACC (100 μM each), or PCIB (all Sigma-Aldrich products). Preliminary tests were performed with concentrations ranging from 1 μM to 1 mM, in order to choose the minimal concentration impacting the ripening kinetics without showing toxic effects. Buffer injection was performed as described previously [35]. Briefly, tomato fruits were infiltrated using a 1 ml syringe with a 0.5 mm needle, inserted 3 to 4 mm into the fruit tissue through the stylar apex. The infiltration solution was gently injected into the fruit until the solution ran off the stylar apex and the hydathodes at the tip of the sepals. Only completely infiltrated fruits were used in the experiments. Controls were treated with buffer only. After the treatment, fruits were incubated in a culture room at 26°C, under 16 h light/8 h dark cycle with a light intensity of 100 μmol s^-1^ m^-2^. After 24 h and 96 h, fruits pericarp was collected and frozen at -80°C until further analysis. For each condition, 27 fruits were sampled arising from 9 different plants.

### Color and pigment measurement

Surface color was assessed with a Chromameter (CR400, Konica Minolta), using the D65 illuminant and the L*, a*, b* space, and the data were processed to obtain Hue as previously described [8]. In the culture room, the fruit color was measured after 6 h, 48 h, 96 h and some fruit were kept up to 8 days for assessing this parameter. Three measures were taken at the equator of each fruit, before being averaged. The Hue angle (in degrees) was calculated according to the following equations: Hue = tan^-1^ (b*/a*) if a > 0 and 180 + tan^-1^ (b*/a*) if a < 0. For pigment analysis, fruit samples were chosen at 96 h after treatment with IAA, ACC, IAA + ACC, PCIB and ground to a fine powder in liquid nitrogen. Pigments (chlorophylls/carotenoids) were extracted from freeze-dried tissues and analyzed as described previously [36] using an Accela U-HPLC system coupled to an Orbitrap high-resolution mass spectrometer (HRMS) operating in positive mode-atmospheric pressure chemical ionization (APCI) (Thermo Fischer Scientific, Waltham, MA).

### ABA and ethylene assays

The ABA assays were performed as described previously [37]. Briefly, 110 mg of frozen tissue, sampled at 96 h after treatments, were extracted at 4°C for 30 min with 400 μl of H_2_O with 10% methanol + 1% acetic acid. The internal standard was [^2^H6] labelled ABA. The extract was centrifuged at 13,000 *g* for 10 min at 4°C. The supernatant was carefully removed and the pellet re-incubated for 30 min with 400 μl of methanol-acetic acid mix. Following the centrifugation, the supernatants were pooled. Extracts were then analysed by LC-MS using an Acquity UPLC coupled to a XevoQtof (Waters, Massachusetts, USA). Analysis parameters were described in Jaulneau et al. [38]. Fruit ethylene production was assayed as previously described [36]. The fruit ethylene production was measured after 6 h, 48 h, 96 h and some fruit were kept up to 8 days in the culture room for assessing this parameter.

### RNA isolation and quantitative PCR (qPCR)

Total fruit RNA was extracted using the PureLink™ Plant RNA Reagent (Invitrogen) according to the manufacturer’s instructions. On fruit sampled at 24 and 96 h, total RNA was treated by DNase I to remove any genomic DNA contamination. First-strand cDNA was reverse transcribed from 2 μg of total RNA using an Omniscript kit (Qiagen). qPCR analyses were performed as previously described [39]. The primer sequences are listed in Additional file 3: Table S1. Relative fold changes were calculated using *SI-actin* as housekeeping gene. As for pigment analyses, three independent RNA isolations were used for cDNA synthesis. Efficiency of DNAse was assessed by PCR with actin primers designed on both size of a zone with an intron, thus giving two bands if genomic DNA is still present.

### Factorial analyses of correspondence, correlation networks and statistics

We used transcript accumulation relative to controls under the ∆∆Ct format to get only positive values, and the carotenoid accumulation levels were calculated relative to controls. The factorial analyses of correspondence were calculated with the explore.xla Excel macro developed previously [40]. Correlation networks were built as previously described [31]. Networks were visualized as organic layouts with Cytoscape version 2.6.3 (www.cytoscape.org) [32]. When LSD are presented they were calculated using Tukey’s HSD.

## Additional files

Additional file 1: Figure S1. Modulation of *ggps* transcript accumulation, by various hormonal treatments, 24th or 96h after treatment (see abbreviations in legend of Figure 2), n=3 biological replicates, bars = std errors. Expression relative to controls (set at 0). Additional file 2: Table S2. Node strengths (ns) of the network in Figure 10, calculated as avg(|ρ|) (Diretto et al, 2010). Additional file 3: Table S1. List of primers used in qPCR experiments.

## Abbreviations

ABA: Abscisic acid
ACC: Aminocyclopropane carboxylic acid
CHLASE: Chlorophyllases
CRTISO: Carotenoid isomerase
CRTR-β: β-carotene hydroxylase
IAA: Indole acetic acid
IPP: Isopentenyl diphosphate
GGPS: GGPP synthase
GGPP: Geranyl-geranyl pyrophosphate
ε-LCY: Lycopene ε-cyclase
β-LCY: Lycopene β-cyclase
NXD: Neoxanthin synthase
PCIB: *p*-chlorophenoxy isobutyric acid
PDS: Phytoene desaturase
PSY: Phytoene synthase
ZEP: Zeaxanthin epoxydase
ZDS: Zeta-carotene desaturase
ZISO: Zeta-carotene isomerase*

## References

[1] Tromas A, Perrot-Rechenmann C (2010). Recent progress in auxin biology. Comptes Rendus Biologies.

[2] Giovannoni JJ (2007). Fruit ripening mutants yield insights into ripening control. Current Opinion Plant Biology.

[3] Cohen J (1996). In vitro tomato fruit cultures demonstrate a role for indole-3-acetic acid in regulating fruit ripening. J Am Soc Hortic Sci.

[4] Davies C, Boss P, Robinson S (1997). Treatment of grape berries, a nonclimacteric fruit with a synthetic auxin, retards ripening and alters the expression of developmentally regulated genes. Plant Physiol.

[5] Given N, Venis M, Grierson D (1988). Hormonal regulation of ripening in the strawberry, a non-climacteric fruit. Planta.

[6] Vendrell M (1985). Dual effect of 2,4-D on ethylene production and ripening of tomato fruit tissue. Physiol Plant.

[7] Muday GK, Rahman A, Binder BM (2012). Auxin and ethylene: collaborators or competitors?. Trends Plant Science.

[8] Ecarnot M, Baczyk P, Tessarotto L, Chervin C (2013). Rapid phenotyping of the tomato fruit model, Micro-Tom, with a portable VIS-NIR spectrometer. Plant Physiol Biochem.

[9] Fraser PD, Truesdale MR, Bird CR, Schuch W, Bramley PM (1994). Carotenoid biosynthesis during tomato fruit development evidence for tissue-specific gene expression. Plant Physiol.

[10] Egea I, Bian W, Barsan C, Jauneau A, Pech JC, Latche A (2011). Chloroplast-to-chromoplast transition in tomato fruit: spectral confocal microscopy analyses of carotenoids and chlorophylls in isolated plastids and time-lapse recording on intact live tissue. Ann Bot.

[11] Hirschberg J (2001). Carotenoid biosynthesis in flowering plants. Current Opinion Plant Biology.

[12] Giuliano G (2014). Plant carotenoids: genomics meets multi-gene engineering. Current Opinion Plant Biology.

[13] Ji K, Kai W, Zhao B, Sun Y, Yuan B, Dai S (2014). SlNCED1 and SlCYP707A2: key genes involved in ABA metabolism during tomato fruit ripening. J Exp Bot.

[14] Giuliano G, Bartley GE, Scolnik PA (1993). Regulation of carotenoid biosynthesis during tomato development. The Plant Cell.

[15] Fantini E, Falcone G, Frusciante S, Giliberto L, Giuliano G (2013). Dissection of tomato lycopene biosynthesis through virus-induced gene silencing. Plant Physiol.

[16] Martel C, Vrebalov J, Tafelmeyer P, Giovannoni JJ (2011). The tomato MADS-box transcription factor RIPENING INHIBITOR interacts with promoters involved in numerous ripening processes in a COLORLESS NONRIPENING-dependent manner. Plant Physiol.

[17] Fujisawa M, Nakano T, Shima Y, Ito Y (2013). A large-scale identification of direct targets of the tomato MADS box transcription factor RIPENING INHIBITOR reveals the regulation of fruit ripening. The Plant Cell.

[18] Ronen G, Cohen M, Zamir D, Hirschberg J (1999). Regulation of caroteniod biosynthesis during tomato fruit development: expression of the gene for lycopene epsilon-cyclase is down-regulated during ripening and is elevated in the mutant Delta. The Plant Journal.

[19] Ronen G, Carmel-Goren L, Zamir D, Hirschberg J (2000). An alternative pathway to beta -carotene formation in plant chromoplasts discovered by map-based cloning of beta and old-gold color mutations in tomato. ProcNatlAcadSci U S A.

[20] Rosati C, Aquilani R, Dharmapuri S, Pallara P, Marusic C, Tavazza R (2000). Metabolic engineering of beta-carotene and lycopene content in tomato fruit. The Plant Journal.

[21] Ma C, Ma B, He J, Hao Q, Lu X, Wang L (2011). Regulation of carotenoid content in tomato by silencing of lycopene β/ε-cyclase genes. Plant Mol Biol Report.

[22] Oono Y, Ooura C, Rahman A, Aspuria ET, Hayashi K, Tanaka A (2003). p-Chlorophenoxyisobutyric acid impairs auxin response in Arabidopsis root. Plant Physiol.

[23] Liotenberg S, North H, Marion-Poll A (1999). Molecular biology and regulation of abscisic acid biosynthesis in plants. Plant Physiol Biochem.

[24] Taylor IB, Burbidge A, Thompson AJ (2000). Control of abscisic acid synthesis. J Exp Bot.

[25] Zhang M, Yuan B, Leng P (2009). The role of ABA in triggering ethylene biosynthesis and ripening of tomato fruit. J Exp Bot.

[26] Galpaz N, Wang Q, Menda N, Zamir D, Hirschberg J (2008). Abscisic acid deficiency in the tomato mutant high-pigment 3 leading to increased plastid number and higher fruit lycopene content. The Plant Journal.

[27] Saltveit ME (1999). Effect of ethylene on quality of fresh fruits and vegetables. Postharvest Biology and Technology.

[28] Frenkel C, Garrison SA (1976). Initiation of lycopene synthesis in the tomato mutant rin as influenced by oxygen and ethylene interactions. HortSci.

[29] Jacob-Wilk D, Holland D, Goldschmidt EE, Riov J, Eyal Y (1999). Chlorophyll breakdown by chlorophyllase: isolation and functional expression of the Chlase1 gene from ethylene-treated Citrus fruit and its regulation during development. The Plant Journal.

[30] Harpaz-Saad S, Azoulay T, Arazi T, Ben-Yaakov E, Mett A, Shiboleth YM (2007). Chlorophyllase is a rate-limiting enzyme in chlorophyll catabolism and is posttranslationally regulated. The Plant Cell.

[31] Diretto G, Al-Babili S, Tavazza R, Scossa F, Papacchioli V, Migliore M (2010). Transcriptional-metabolic networks in β-carotene-enriched potato tubers: the long and winding road to the golden phenotype. Plant Physiol.

[32] Cline MS, Smoot M, Cerami E, Kuchinsky A, Landys N, Workman C (2007). Integration of biological networks and gene expression data using Cytoscape. Nat Protoc.

[33] Neuman H, Galpaz N, Cunningham FX, Zamir D, Hirschberg J (2014). The tomato mutation nxd1 reveals a gene necessary for neoxanthin biosynthesis and demonstrates that violaxanthin is a sufficient precursor for abscisic acid biosynthesis. The Plant Journal.

[34] Pirrello J, Jaimes-Miranda F, Sanchez-Ballesta MT, Tournier B, Khalil-Ahmad Q, Regad F (2006). Sl-ERF2, a tomato ethylene response factor involved in ethylene response and seed germination. Plant Cell Physiol.

[35] Orzaez D, Mirabel S, Wieland WH, Granell A (2006). Agroinjection of tomato fruits. A tool for rapid functional analysis of transgenes directly in fruit. Plant Physiol.

[36] Liu M, Diretto G, Pirrello J, Roustan JP, Li Z, Giuliano G (2014). The chimeric repressor version of an Ethylene Response Factor (ERF) family member, Sl-ERF.B3, shows contrasting effects on tomato fruit ripening. New Phytol.

[37] Forcat S, Bennett MH, Mansfield JW, Grant MR (2008). A rapid and robust method for simultaneously measuring changes in the phytohormones ABA, JA and SA in plants following biotic and abiotic stress. Plant Methods.

[38] Jaulneau V, Lafitte C, Jacquet C, Fournier S, Salamagne S, Briand X (2010). Ulvan, a sulfated polysaccharide from green algae, activates plant immunity through the jasmonic acid signaling pathway. J Biomed Biotechnol.

[39] Chervin C, Deluc L (2010). Ethylene signalling receptors and transcription factors over the grape berry development: gene expression profiling. Vitis.

[40] Vaillé J. La Statistique au service des Données: quelques macros Excel pour faire de l’analyse exploratoire des données. 2010, Modulad n°43, http://www.modulad.fr/index.htm

[41] Bramley PM (2002). Regulation of carotenoid formation during tomato fruit ripening and development. J Exp Bot.

