# Supplementary figures and images for: Carotenoid accumulation during tomato fruit ripening is modulated by the auxin-ethylene balance
Source: BMC Plant Biol. 2015 May 8;15:114. doi: 10.1186/s12870-015-0495-4 (PMC4424491; doi:10.1186/s12870-015-0495-4)

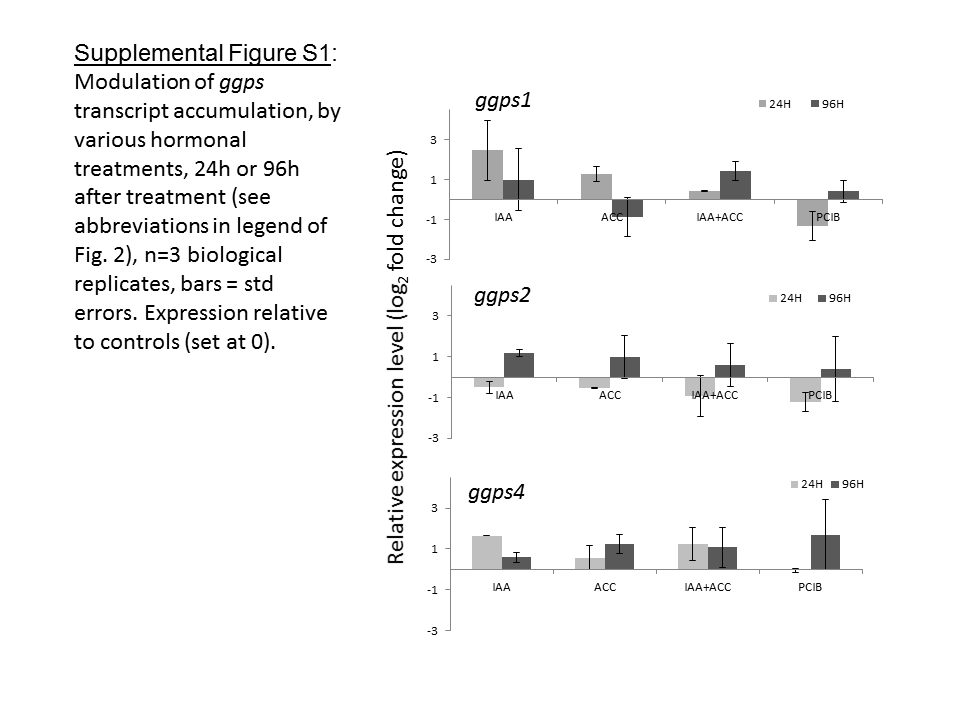

Supplement: Additional file 1: Figure S1. — Modulation of ggps transcript accumulation, by various hormonal treatments, 24th or 96h after treatment (see abbreviations in legend of Figure 2), n=3 biological replicates, bars = std errors. Expression relative to controls (set at 0). [file 12870_2015_495_MOESM1_ESM.tiff]

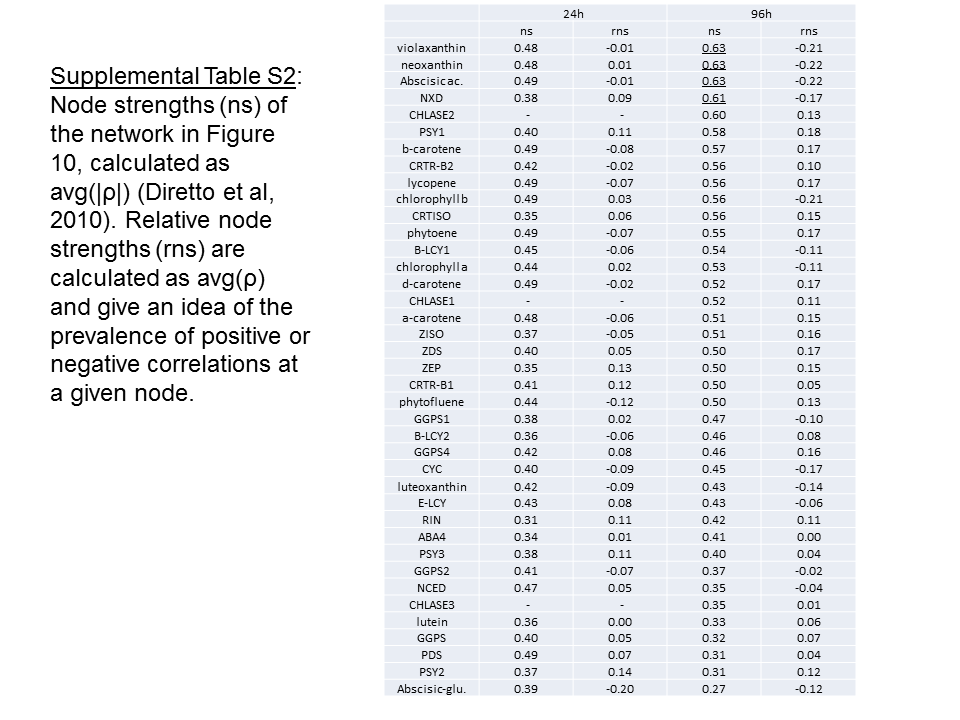

Supplement: Additional file 2: Table S2. — Node strengths (ns) of the network in Figure 10, calculated as avg(|ρ|) (Diretto et al, 2010). [file 12870_2015_495_MOESM2_ESM.tiff]

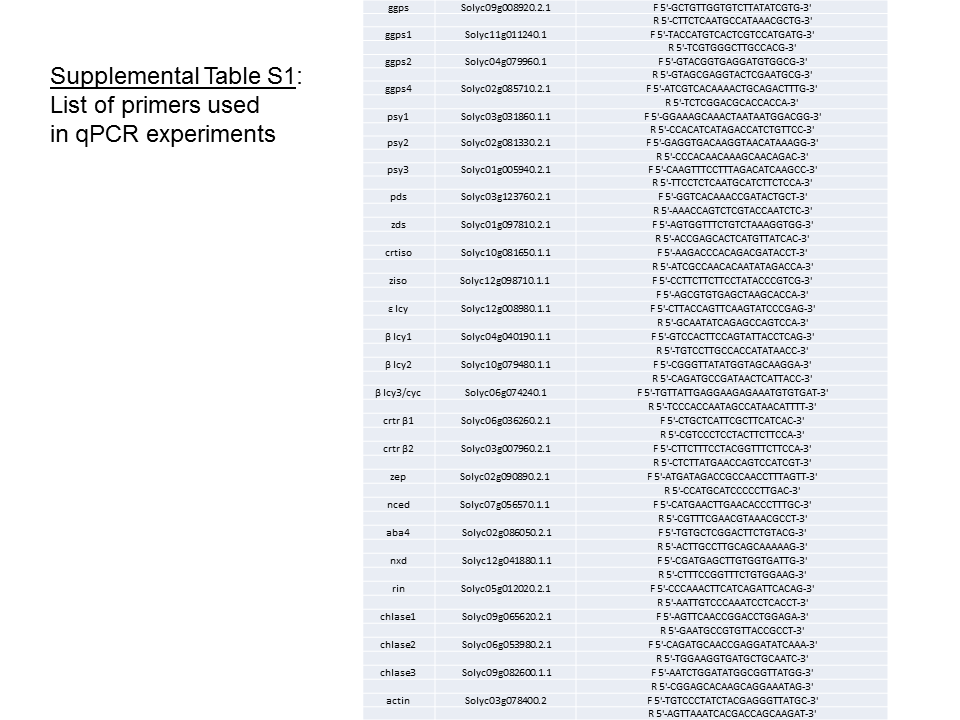

Supplement: Additional file 3: Table S1. — List of primers used in qPCR experiments. [file 12870_2015_495_MOESM3_ESM.tiff]
